# Lymphocyte transformation test in pediatric drug hypersensitivity: case series report from a tertiary care hospital

**DOI:** 10.3389/fimmu.2026.1787795

**Published:** 2026-05-15

**Authors:** Olga Rogozina, Susana Martín-López, Fiorela Cinthia Dueñas López, Zoraida del Solar Moreno, Larry Leiva Castrejon, Daniela Aguilar-Concepción, María Jiménez-González, Ana Martínez Feito, Miguel González-Muñoz, Elena Ramírez

**Affiliations:** 1 Clinical Pharmacology Department, La Paz University Hospital-IdiPAZ, Faculty of Medicine, Autonomous University of Madrid, Madrid, Spain; 2 Immunology Department, La Paz University Hospital-IdiPAZ, Madrid, Spain; 3 Clinical Trial Unit, La Paz University Hospital-IdiPAZ, Madrid, Spain

**Keywords:** adverse drug reaction (ADR), causality algorithm, diagnostic accuracy, drug hypersensitivity, drug provocation test (DPT), lymphocyte transformation test (LTT), pediatrics, pharmacovigilance

## Abstract

**Background:**

Diagnosing adverse drug reactions (ADRs) in children is challenging due to coexisting infections. Reliable *in vitro* methods are critically needed.

**Objectives:**

To evaluate the lymphocyte transformation test (LTT) in supporting the Spanish Pharmacovigilance System (SPS) causality algorithm for identifying culprit drugs in pediatric ADRs, and to estimate the diagnostic accuracy of the SPS algorithm, LTT, skin tests, and drug provocation testing (DPT).

**Methods:**

Retrospective case-series (2018–2024) at a tertiary hospital. Children with adjudicated ADRs were identified through pharmacovigilance programs and clinical referrals. Tolerance upon re-exposure served as the reference standard to estimate sensitivity, specificity, and predictive values.

**Results:**

We analyzed 81 children (mean age 9.2 ± 5.9 years; 46.9% female). Most had no prior drug allergies (88.9%). The ADR spectrum was primarily cutaneous and hepatic (rash 19.5%, urticaria 13.0%, liver injury 11.9%). Frequent culprit drugs included amoxicillin (8.4%), paracetamol (7.9%), amoxicillin/clavulanic acid (6.9%), and ibuprofen (6.4%). Using re-exposure as the reference standard, LTT demonstrated 100% sensitivity (95% CI: 51.0-100%) and 100% NPV (95% CI: 89.3-100%), with 78% specificity (95% CI: 12.6-57.6%). Significant associations were found between LTT positivity and SPS scores ≥4 (p<0.001), and between LTT and DPT outcomes (p=0.008).

**Conclusion:**

LTT positivity correlates significantly with high causality scores and DPT outcomes in children. LTT is a valuable, non-invasive tool for evaluating T-cell–mediated reactions, particularly when DPT is risky or contraindicated.

## Introduction

The World Health Organization (WHO) defines adverse drug reaction (ADR) as “a response to a medicine which is noxious and unintended, and which occurs at doses normally used in man” (1). ADRs in children significantly influence illness, death rates, and medical costs (2–4). Reported ADR rates range from 1.5–18.1% in hospitals and 0.7–2.74% in outpatient care, with 0.2–5.8% leading to hospitalization (5). Children represent 7.7% of all reports received by the Uppsala Monitoring Centre (6). The medications associated with ADRs differ from those in adults (7, 8). In pediatrics, beta-lactam antibiotics are most frequently implicated, followed by NSAIDs and other non-beta-lactam antibiotics (9). Diagnosing ADRs is particularly challenging in this population, as symptoms often reflect coexisting infections rather than true hypersensitivity (10, 11). The diagnostic approach is similar to that in adults and comprises a thorough clinical history, skin testing, validated laboratory investigations when available, and drug provocation testing (12).

*In vivo* testing is typically the first-line diagnostic approach for both immediate and non-immediate drug hypersensitivity reactions in children (11, 13). Skin tests, including skin prick tests (SPT), intradermal tests (IDT), and patch tests, demonstrate low sensitivity for non-immediate reactions in the pediatric population (14–19).

Due to the limited sensitivity of skin tests in delayed reactions and the potential severity of these events, drug provocation testing (DPT) remains the gold standard (20). Nevertheless, DPT carries inherent risks and is often contraindicated in severe drug reactions. In recent years, risk stratification has become a key approach to tailoring the diagnostic strategy according to the perceived risk, while ensuring a high level of patient safety. Skin testing is recommended prior to DPT, although it may be omitted in patients classified as low risk. The task force proposes a clear definition of such low-risk profiles in both pediatric and adult populations (21).

Consequently, there is a critical need to develop reliable *in vitro* diagnostic methods with adequate sensitivity and specificity, particularly to reduce reliance on provocation testing in children, who may be less tolerant of invasive procedures (11).

Current *in vitro* diagnostic tools, such as the lymphocyte transformation test (LTT), is a laboratory test that can be used to help diagnose serious ADRs. The LTT measures the response of white blood cells (lymphocytes) to a drug that is suspected of causing the ADR. If the lymphocytes show a significant increase in response to the drug, then this is a strong indication that the drug is the cause of the ADR (22, 23). Nevertheless, the test still remains largely restricted to research contexts due to inconsistent sensitivity and limited clinical validation (19). According to the official position of the ENDA/EAACI Drug Allergy Interest Group, the sensitivity and specificity of the LTT vary widely from 27% to 88.8% and 63% to 100%, respectively, depending on the implicated drug and clinical presentation. Higher diagnostic performance has been reported for beta-lactams and anticonvulsants, particularly in conditions such as maculopapular exanthema (MPE), fixed drug eruption (FDE), acute generalized exanthematous pustulosis (AGEP), and DRESS syndrome. However, its utility is limited in severe reactions like Stevens-Johnson syndrome (SJS) and toxic epidermal necrolysis (TEN) (20).

Several unmet needs remain in this field, including the necessity to conduct large-scale studies in well-characterized patients confirmed through skin testing and drug provocation tests. Also, it is necessary to establish robust estimates of sensitivity, specificity, and predictive values for current *in vitro* approaches, such as LTT.

The main objective of this study was to evaluate the usefulness of LTT in supporting the casualty algorithm of the SPS (Spanish Pharmacovigilance System) for diagnosing of the causative drug in ADR in children.

Secondary objectives were to describe ADRs in pediatric patients and evaluate sensitivity, specificity, and predictive values for the following diagnostic tools: SPS algorithm, LTT, SPT, IDT, patch test and DPT.

## Materials and methods

### Setting and patients

This retrospective case series study was conducted at La Paz University Hospital in Madrid, Spain, a tertiary-care teaching facility. Since 2007, all admissions have been monitored by the Proactive Pharmacovigilance Program from Laboratory Signals in Hospital to proactively detect serious ADRs (24).

The study was approved by the La Paz University Hospital Ethics Committee (Code PI-3226; 25 May 2018). Due to the retrospective nature, the study was exempt from informed consent requirements.

Inclusion criteria were ADR-specific and required: (i) fulfillment of the clinical or analytical definition of the relevant adverse drug reaction; (ii) reasonable exclusion of alternative etiologies according to the SPS algorithm (25); and (iii) LTT performed for at least one suspected drug. Medication errors were excluded.

For all patients with suspected adverse drug reaction a complete report was submitted to the pharmacovigilance center in Madrid, Spain (https://www.notificaram.es).

### Case ascertainment and data sources

Cases were identified through three sources. The first source (i) was routine surveillance within the institutional pharmacovigilance program. This procedure has been described previously (24) and comprises three phases: Phase I involved continuous surveillance of laboratory results available in the hospital information system (24 hours per day, seven days per week) to identify abnormal values; Phase II included reconciling patient identifiers to prevent duplicate entries and reviewing electronic medical records to exclude abnormalities clearly attributable to non-drug etiologies; and *Phase III*, in which remaining cases underwent individualized evaluation. The other sources included (ii) inter-specialty consultations managed by the Pharmacovigilance Unit of the Clinical Pharmacology Department; and (iii) review of data collected by the Immunology Department, including results of LTT, SPT, IDT, patch tests, and DPT, which were requested by the Allergy Department as part of the patients’ allergy study.

Since cases were identified from existing diagnostic records and validated pharmacovigilance reports, all included patients had complete clinical and laboratory datasets required for analysis. Data were collected retrospectively between 2018 and 2024 by reviewing the institutional electronic medical records (EMR). For each case, the index date was defined as the earliest documentation of either symptom onset or an abnormal laboratory result. Any previously unassessed ADR identified during history review was recorded as a separate case.

Data were managed using an electronic Case Report Form (eCRF) developed in the RedCap (Research Electronic Data Capture) platform. Summary of Collected Variables.

For each patient, the following data were systematically recorded in the database:

- Demographics and Clinical History: age, sex, number of comorbidities or chronic conditions, and previous history of allergic reactions or ADRs.
- Laboratory and Microbiological Tests: Results from blood tests and serologies (e.g., EBV, HHV-6, or Mycoplasma) to identify potential cofactors or alternative etiologies.
- ADR Characterization: type of reaction (ICD-10), clinical symptoms (e.g., pruritus, fever, mucosal lesions), anatomical location of skin lesions, and time to symptom resolution. To ensure diagnostic consistency and align with international standards, clinical manifestations were coded according to the International Classification of Diseases, 10th Revision (ICD-10). Following the recommendations of the ENDA/EAACI Drug Allergy Interest Group, clinical descriptions of ‘macular’, ‘papular’, and ‘maculopapular’ rashes were consolidated into a single category: maculopapular exanthema. Specific ICD-10 codes were assigned to differentiate the extent and nature of reactions:
- Code R21 (Rash and other nonspecific skin eruption): Assigned to non-generalized rashes involving two or fewer body areas, particularly when a viral differential diagnosis was considered.
- Code L27.0 (Generalized skin eruption due to drugs and medicaments taken internally): Applied to widespread reactions affecting three or more body areas with clear clinical indicators of drug involvement.
- Code L27.1 (Localized skin eruption due to drugs and medicaments taken internally): Reserved specifically to denote fixed drug eruptions.

Pharmacological Data and Causality:

- Suspected drugs were identified and classified according to the Anatomical Therapeutic Chemical (ATC) system.
- Causality was evaluated using causality algorithms.

Diagnostic Workup:

- *In vitro*: lymphocyte transformation test (LTT) results, expressed as the stimulation index (SI).
- *In vivo*: results from skin prick tests (SPT), intradermal tests (IDT), patch tests, and drug provocation tests (DPT).

Re-exposure: clinical tolerance data following the re-introduction of the suspected drug.

### Causality assessment

Causality was assessed using the SPS (Spanish Pharmacovigilance System) causality algorithm (25), which evaluates the relationship between a suspected drug and an ADR across seven criteria: latency from treatment initiation to ADR onset; prior evidence in the literature linking the drug to the ADR; evolution after drug withdrawal (de-challenge); effect of re-exposure (re-challenge); presence of alternative, non-drug or other drug–related causes; contributing factors favoring a causal relationship; and results of tests supporting drug causality. The domain-specific score (range, −8 to +11) classifies each drug as definite (≥8), probable (6–7), possible (4–5), conditional (1–3), or unrelated (<0). An SPS score ≥ +4 was considered drug-related.

The causality assessment for drug-induced liver injury was performed using updated RUCAM (Roussel Uclaf Causality Assessment Method), the most employed algorithm for assessing causality in drug-induced liver injury (26, 27). The updated RUCAM algorithm evaluates the same parameters, such as SPS, but specific to drug-induced liver injury. The total score (ranging from −7 to +14) from the domain-specific assessment classifies the drug into 5 separate categories: highly probable (≥9), probable (6–8), possible (3–5), unlikely (1–2) or excluded (<0). A RUCAM score ≥3 was considered drug-related.

In routine clinical practice, application of the causality algorithm is sometimes omitted. For those ADRs in which causality assessment was not performed, we conducted such assessment retrospectively based on the clinical history at the ADR onset, without considering results of laboratory testing, allergy testing and subsequent re-exposure if happened.

Drugs implicated in the causation of ADRs were classified into therapeutic subgroups according to the Anatomical Therapeutic Chemical (ATC) system.

### *In vitro* lymphocyte transformation test

Lymphocyte proliferation was measured as previously described (23, 28). Briefly, mononuclear cells were separated over a density gradient (Histopaque-1077, Sigma-Aldrich) from fresh peripheral blood and were plated in flat bottom wells of microtitre plates at 2×10^5^ cells/well. Cells were incubated for 6 days with various drug concentrations in triplicate. Drugs were assayed at concentrations of 1, 10 and 100 μg/ml, and occasionally, a lower or higher concentration (0.1, 200 or 500 μg) were used, as previously described (23). We used phytohemagglutinin (5 μg/ml) as a positive control. For the final 18 hours of the incubation period, proliferation was determined by adding 1 μCi (3H) thymidine.

Proliferative responses were expressed as the stimulation index (SI), calculated as the ratio between the mean counts per minute in cultures with the drug and those without drug. An LTT result was considered positive if the SI was ≥2 at any tested drug concentration, as this threshold is the most widely accepted in clinical and research practice. To improve interpretability, we applied the binary classification positive/negative across all cases.

### *In vivo* testing

Clinical history alone is insufficient to establish the diagnosis of ADRs, and additional testing is often required (29). In pediatrics, SPT, IDT and patch are clinically validated tools, DPT remains the diagnostic gold standard, particularly for confirming tolerance in non-severe reactions where risk stratification supports its use for delabeling (21, 30). The choice of diagnostic tool was individualized according to the implicated drug and the severity of the reaction phenotype (risk stratification); consequently, not all patients underwent the same battery of tests. For each case, we recorded the tests performed as determined by routine clinical practice. All procedures were conducted in accordance with published practice guidelines that prioritize safety profiles and distinguish between non-severe (low-risk) and severe (high-risk) approaches (21, 31–34).

### Statistical analysis

Continuous variables are presented as mean ± standard deviation (SD) or median with interquartile range (IQR), depending on normality assessed by the Kolmogorov-Smirnov test. Categorical variables are expressed in absolute terms and percentages. We employed the chi-squared test to compare the categorical variables and employed Student’s t-test for the continuous variables with a normal distribution. In the event the data did not have a normal distribution, we used the nonparametric Mann-Whitney *U*-test or Kruskal Wallis test, as appropriate. Differences were considered significant when the *p*-value was <0.05. For comparisons involving prick, patch, and intradermal skin tests, any 2×2 table with a zero cell was adjusted using Haldane’s continuity correction (adding 0.5 to each cell). From these corrected tables, we then calculated the odds ratios (OR), sensitivity, specificity, positive predictive value (PPV), and negative predictive value (NPV), each with its 95% confidence interval (CI). To evaluate the concordance between paired results from different diagnostic tests (e.g., LTT vs. DPT), McNemar’s test was employed. A p-value < 0.05 was considered statistically significant. Descriptive analyses were performed using R (Integrated Development for R Studio, PBC, Boston, MA; http://www.rstudio.com, accessed on 19 February 2025). A formal sample size calculation was not performed because this study was designed as a descriptive case series. The sample size was determined by the total number of pediatric patients who met the inclusion criteria and underwent LTT testing at our tertiary care hospital during the study period. This approach provides a representative reflection of real-world clinical practice in our setting. Regarding missing data, a complete-case analysis approach was adopted. Variables with missing values, such as specific allergy test results or clinical parameters not documented in the electronic medical records, were excluded from the specific calculations where they were required. No imputation methods were used, as the study relied on existing clinical documentation from routine practice.

## Results

### Characteristics of patients and ADR episodes

We analyzed 81 patients (46.9% female; 53.1% male) with a median age of 11 [4-15] years. Comorbidity burden was modest (median 2 [1-3.75]); 28.4% had no chronic conditions, 38.3% had one, and 33.3% had ≥ two. Prior allergy history (including ADRs) was uncommon (11.1%). While the study included 81 patients, these individuals experienced a total of 92 distinct ADR episodes (cases). Most patients experienced a single ADR episode (85.0%); 14.8% had multiple events (median number of ADRs 1[1-1]). To reflect the complete clinical spectrum, all subsequent characteristics regarding symptoms, treatments, and diagnostic findings are reported based on the total number of 92 episodes. Patient characteristics and the clinical spectrum of these ADR episodes are summarized in **Table 1**.

**Table 1:** Demographic characteristics of patients and clinical spectrum of pediatric ADR.

Variable	Results
Sex, n (%)	
Female	38 (46.9)
Overall mean age (median [IQR])	11 [4-15]
Chronic conditions, n (%)	
No chronic conditions	23 (28.4)
1	31 (38.3)
2 or more	27 (33.3)
Number of comorbidities (median [IQR])	2 [1-3.75]
History of allergic reactions (including ADRs), n (%)	
No	72 (88.9)
ADR, n (%)	
1	69 (85)
2	8 (9.8)
3	3 (3.7)
4	1 (1.2)
Number of ADR (median [IQR])	1[1-1]
ADR types, n (%)	
1. Rash and other nonspecific skin eruptions	18 (19.5)
2. Urticaria	12 (13.0)
3. Drug-induced liver injury	11 (11.9)
4. Generalized skin eruption due to drugs and medicaments taken internally	9 (9.8)
5. Drug rash with eosinophilia and systemic symptoms syndrome	8 (8.7)
6. Agranulocytosis	8 (8.7)
7. Tubulo-interstitial nephritis	7 (7.6)
8. Localized skin eruption due to drugs and medicaments taken internally	6 (6.5)
9. Drug-induced acute pancreatitis	(3.3)
10. Eosinophilia	2 (2.2)
11. Encephalitis, myelitis and encephalomyelitis	2 (2.2)
12. Acute myocarditis	2 (2.2)
13. Toxic erythema	1 (1.1)
14. Thrombocytopenia	1 (1.1)
15. Aplastic anemia	1 (1.1)
16. Angioneurotic oedema	1 (1.1)
Symptoms, n (%)	
Yes	79 (86.0)
Number of symptoms (median [IQR])	2 [1-3]
Type of symptom, n (%)	
➢ Skin lesion	56 (70.0)
➢ Pruritus	36 (45.0)
➢ Fever	31 (38.8)
➢ Angioedema	12 (15)
➢Nausea/Vomiting	11 (13.8)
➢ Abdominal pain	9 (11.3)
➢General malaise	9 (11.3)
➢ Weakness	8 (10)
➢ Mucosal lesion	7 (8.8)
➢ Cough	4 (5.0)
➢ Other minor symptoms	25 (31.7)
Type of skin lesion, n (%)	
➢Maculopapular exantema	37 (51.4)
➢ Generalized skin rash	19 (26.4)
➢ Urticaria	16 (22.2)
Location of skin lesion, n (%)	
➢ Lower extremity	42 (75.0)
➢ Upper extremity	41 (73.2)
➢ Chest	36 (64.3)
➢ Face	34 (60.7)
➢ Abdomen	30 (53.6)
➢ Back	20 (35.7)
➢ Neck	15 (26.8)
➢ Genitals	9 (16.1)
Treatment received, n (%)	
Yes	68 (73.9)
Number of administered medications (median [IQR])	2 [1-2]
Treatment administered, n (%)	
➢ Corticosteroids	54 (44.6)
➢Antihistaminic drugs	35 (29.0)
➢ Paracetamol	8 (6.6)
➢ NSAIDs	4 (3.3)
➢ Other	20 (16.5)
➢ Time to LTT (median [IQR])	176 [117-324]

ADR, adverse drug reaction; IQR, interquartile range; LTT, lymphocyte transformation test; n, number of patients/events; NSAIDs, non-steroidal anti-inflammatory drugs. Data are expressed as absolute frequency (percentage) for categorical variables, and as median [interquartile range] for continuous variables.

The ADR spectrum was predominantly cutaneous and hepatic: nonspecific rash (19.5%) and urticaria (13.0%) were most frequent, followed by drug-induced liver injury (11.9%), generalized drug eruptions (9.8%), DRESS (8.7%), agranulocytosis (8.7%), and tubulo-interstitial nephritis (7.6%). Symptoms were reported by 86.0% (median 2 [1-3] across the cohort); among symptomatic patients (n=79), 48.1% had ≥3 symptoms. The leading manifestations were skin lesions (70.0%), pruritus (45.0%), and fever (38.8%). Among those with skin involvement, maculopapular exantema (51.4%), generalized rash (26.4%), and urticaria (22.0%) predominated; lesions most often affected the lower (75.0%) and upper (73.2%) extremities, chest (64.3%), face (60.7%), and abdomen (53.6%).

Treatment was administered to 73.9% of patients [median 2 (1-2) medications among treated]. Antihistamines and corticosteroids were most used, with 44.6% and 29.0%, respectively. Regarding the diagnostic workup, laboratory testing frequently showed abnormalities (blood tests positive in 58.7%). Microbiological testing yielded positive finding in 28.2% of results. These results were predominantly driven by serological evidence of pathogens such as human herpesvirus 6, *Mycoplasma*, and Epstein Barr virus, alongside various culture and PCR findings. Comprehensive data detailing these laboratory and microbiological results are provides in the **Supplementary Material** (**Supplementary Table 1**). The median time to LTT was 176 [117-324]days. The marked variability reflects that some patients reported ADR manifestations years earlier that had not been investigated at the time, and these historical episodes were recorded as separate events. Consequently, the dataset includes numerous outliers representing multi-year delays before LTT was performed.

Symptom resolution times varied widely. Systemic events had the longest mean durations - agranulocytosis (62.5 ± 104.8 days), drug-induced liver injury (54.5 ± 81.3), and acute myocarditis (53.0 ± 72.1) - whereas most cutaneous or immunoallergic reactions resolved within two weeks (e.g., DRESS, 14.1 ± 11.1), with rapid recovery (≤1 week) for toxic erythema, localized eruptions, and angioedema. Complete list of recovery times is listed in **Table 2**.

**Table 2:** ADR symptoms resolution time.

ADR type	Symptoms resolution time, days, mean (SD)
Agranulocytosis	62.5 (104.8)
Drug-induced liver injury	54.5 (81.3)
Acute myocarditis	53.0 (72.1)
Generalized skin eruption due to drugs and medicaments taken internally	31.9 (26.2)
Drug rash with eosinophilia and systemic symptoms syndrome	14.1 (11.1)
Rash and other nonspecific skin eruptions	13.8 (17.9)
Eosinophilia	13.5 (0.7)
Urticaria	12.8 (21.8)
Tubulo-interstitial nephritis	12.4 (12.2)
Thrombocytopenia	10.0 (0.0)
Encephalitis, myelitis and encephalomyelitis	10.0 (7.1)
Drug-induced acute pancreatitis	9.3 (4.0)
Toxic erythema	7.0 (0.0)
Localized skin eruption due to drugs and medicaments taken internally	4.3 (2.9)
Angioneurotic oedema	4.0 (0.0)
Aplastic anaemia	NA

NA, Not Available.

### Culprit drugs

A total of 202 drugs were evaluated across the 92 ADR episodes documented in this study. Most ADR episodes involved one to two suspected agents (41.3% and 32.6%, respectively); up to seven were assessed in some cases. The mean number of drugs evaluated per episode was 2.2 ± 1.5. The most frequently evaluated molecules were amoxicillin (17; 8.4%), paracetamol (16; 7.9%), amoxicillin/clavulanic acid (14; 6.9%), ibuprofen (13; 6.4%), other antiviral vaccines (12; 5.9%), and metamizole (10; 5.0%). Together with cefotaxime and meropenem (7 each; 3.5%), TMP–SMX (6; 3.0%), and levetiracetam (5; 2.5%), the top ten agents accounted for approximately 53% of all suspected exposures. By class, β-lactams (penicillins/cephalosporins/carbapenems, including meropenem) comprised 28% (57/202), while analgesics/antipyretics and NSAIDs (paracetamol, ibuprofen, metamizole, naproxen, dexketoprofen, indomethacin, tramadol) comprised 23% (47/202). A detailed breakdown of phenotype-drug associations (n, % within ADR subtype) is provided in the **Supplementary Material** (**Supplementary Table 2**).

### Drug subgroup implication and LTT positivity

Across 175 drug evaluations coded by ATC, antibacterial drugs (J01) were the most frequently assessed class (81/175; 46.3%), followed by analgesic drugs (N02; 14.3%) and anti-inflammatory and antirheumatic drugs (M01; 10.3%) (**Table 3**). The mean SPS causality score across all classes was 5.2 ± 2.1. Among higher-volume classes, mean SPS values were 5.6 ± 2.2 for antibacterial drugs (J01), 5.4 ± 1.7 for anti-inflammatory and antirheumatic drugs (M01), and 4.5 ± 2.8 for analgesic drugs (N02).

**Table 3:** Number of drugs, SPS score and number of positive LTTs by ATC groups.

Drug subgroups (ATC classification)	Drugs, n (%)	SPS score [Mean (SD)]	No. of positive LTTs per subgroup/total no. of positive LTTs, n (%)
J01 - Antibacterial drugs	81 (46.3)	5.6 (2.2)	41/66 (62.1)
N02 - Analgesic drugs	25 (14.3)	4.5 (2.8)	4/66 (6.1)
M01 - Anti-inflammatory and antirheumatic drugs	18 (10.3)	5.4 (1.7)	5/66 (7.6)
J07 - Vaccines	9 (5.1)	4.8 (1.3)	1/66 (1.5)
N03 - Antiepileptic drugs	9 (5.1)	5.9 (1.1)	7/66 (10.6)
J04 - Antimycobacterials	5 (2.9)	5.0 (2.8)	1/66 (1.5)
A02 - Drugs for acid related disorders	4 (2.3)	5.0 (0.0)	0 (0.0)
J02 - Antimycotic drugs	4 (2.3)	5.0 (0.8)	1/66 (1.5)
L04 - Immunosuppressants drugs	4 (2.3)	3.8 (1.3)	2/66 (3.0)
M03 - Muscle relaxants	3 (1.7)	5.0 (1.7)	1/66 (1.5)
J05 - Antiviral drugs	2 (1.1)	5.5 (0.7)	0 (0.0)
A04 - Antiemetics and antinauseants	1 (0.6)	6	1/66 (1.5)
A07 - Antidiarrheals, intestinal anti-inflammatory/anti-infective agents	1 (0.6)	7	0 (0.0)
C10 - Lipid modifying agents	1 (0.6)	3	0 (0.0)
M04 - Antigout preparations	1 (0.6)	6	0 (0.0)
N01 - Anesthetic drugs	1 (0.6)	7	1/66 (1.5)
N05 - Psycholeptics drugs	1 (0.6)	5	0 (0.0)
P02 - Anthelmintic drugs	1 (0.6)	2	0 (0.0)
R05 - Cough and cold drugs	1 (0.6)	3	0 (0.0)
R06 - Antihistamines for systemic use	1 (0.6)	4	1/66 (1.5)
S01 - Ophthalmological drugs	1 (0.6)	2	ND
V03 - All other therapeutic products	1 (0.6)	1	ND
Total	175 (100.0)	5.2 (2.1)	66 (100.0)

ND, Not Done.

Of 66 positives LTTs, antibacterial drugs (J01) accounted for 62.1% (41/66), indicating an over-representation relative to their exposure share. Antiepileptic drugs (N03) contributed 10.6% (7/66) of all positive results despite only 5.1% of tested drugs, whereas analgesic drugs (N02) contributed 6.1% (4/66) despite 14.3% of exposures. Other categories, such as vaccines (J07), antimycotics (J02), and muscle relaxants (M03), showed minor contributions to overall positivity (1.5% each). Immunosuppressant drugs (L04) showed a comparatively low mean SPS (3.8 ± 1.3) and 3.0% of positives.

Suspected exposures clustered in antibacterials and, to a lesser extent, analgesic/anti-inflammatory agents. LTT positivity was concentrated in antibacterials and antiepileptics, consistent with their higher SPS or known immunoallergenic potential; analgesics and vaccines had lower yields.

### Relation among ADR type, SPS score and LTT result

Across all phenotypes, the mean SPS causality score was 6.0 ± 1.7 and 77/202 drugs (38.1%) yielded a positive LTT (**Table 4**). LTT positivity was highest among the more prevalent cutaneous and hepatic phenotypes: generalized drug eruption (7/15; 46.7%), DRESS (12/26; 46.2%), localized drug eruption (5/11; 45.5%), rash (nonspecific) (18/43; 41.9%), and drug-induced liver injury (11/27; 40.7%). Moderate yields were observed in tubulo-interstitial nephritis (TIN) (7/21; 33.3%) and urticaria (6/18; 33.3%).

**Table 4:** Number of drugs, SPS score and number of positive LTTs by ADR type.

ADR type	Drugs, n (%)	SPS score [Mean (SD)]	No. of positive LTTs per ADR/total no. of drugs studied per ADR, n (%)
Rash and other nonspecific skin eruptions	43 (21.3)	6.0 (1.6)	18/43 (41.9)
Drug-induced liver injury	27 (13.4)	N/A	11/27 (40.7)
Drug rash with eosinophilia and systemic symptoms syndrome	26 (12.9)	6.0 (2.8)	12/26 (46.2)
Tubulo-interstitial nephritis	21 (10.4)	6.0 (1.7)	7/21 (33.3)
Urticaria	18 (8.9)	6.6 (1.2)	6/18 (33.3)
Agranulocytosis	17 (8.4)	4.9 (1.6)	4/17 (23.5)
Generalized skin eruption due to drugs and medicaments taken internally	15 (7.4)	6.4 (2.5)	7/15 (46.7)
Localized skin eruption due to drugs and medicaments taken internally	11 (5.4)	6.0 (0.6)	5/11 (45.5)
Encephalitis, myelitis and encephalomyelitis	5 (2.5)	5.5 (2.1)	0 (0.0)
Acute myocarditis	4 (2.0)	6.0 (0.0)	0 (0.0)
Eosinophilia	4 (2.0)	5.0 (1.4)	1/4 (25.0)
Drug-induced acute pancreatitis	4 (2.0)	7.0 (0.0)	2/4 (50.0)
Toxic erythema	2 (1.0)	7.0	1/2 (50.0)
Aplastic anaemia	2 (1.0)	3.0	0 (0.0)
Thrombocytopenia	2 (1.0)	6.0	2/2 (100.0)
Angioneurotic oedema	1 (0.5)	6.0	1/1 (100.0)
Total	202 (100.0)	6.0 (1.7)	77

N/A, Not applicable.

Regarding hematological and organ-specific reactions, results were heterogeneous. Agranulocytosis showed the lowest mean SPS (4.9 ± 1.6) and a correspondingly low LTT positivity (4/17; 23.5%), and no positives were seen for encephalitis/myelitis (0/5) or acute myocarditis (0/4). Very high proportions in thrombocytopenia (2/2; 100%) and angioneurotic oedema (1/1; 100%) should be interpreted cautiously given the very small sample sizes (n=2 and n=1, respectively). High SPS scores were also recorded for acute pancreatitis and toxic erythema (7.0 each; both small n), followed by urticaria (6.6 ± 1.2), generalized drug eruption (6.4 ± 2.5), and rash/localized eruption/tubulo-interstitial nephritis (each 6.0).

Per case, a mean of 2.2 ± 1.5 drugs was assessed, of which 1.8 ± 1.3 met SPS ≥ 4 (**Table 5**). Overall, 51/81 cases (63.0%) had ≥1 positive LTT. Polyexposure was greatest in DRESS (3.3 ± 2.4 drugs/case) and tubulo-interstitial nephritis (3.0 ± 1.5), and lowest in acute pancreatitis (1.3 ± 0.6) and urticaria (1.5 ± 0.7). The probability of at least one positive LTT per case was highest for cutaneous phenotypes: DRESS (7/8; 87.5%), rash (14/18; 77.8%), generalized eruption (6/9; 66.7%), and localized eruption (4/6; 66.7%). Tubulo-interstitial nephritis also showed a high case-level yield (5/7; 71.4%). By contrast, acute myocarditis and encephalitis/myelitis had 0/2 positive LTT cases each.

**Table 5:** Number of cases, drugs per case, drugs with SPS score ≥4 and number of positive LTTs by ADR type.

ADR type	No. of cases, n (%)	No. of drugs assessed per case, n (SD)	No. of drugs with SPS≥4, n (SD)	No. of at least one positive LTT per case/total no. of ADR cases
Rash and other nonspecific skin eruptions	18 (22.2)	2.4 (2.0)	1.9 (1.7)	14/18
Urticaria	12 (14.8)	1.5 (0.7)	1.3 (0.6)	5/12
Generalized skin eruption due to drugs and medicaments taken internally	9 (11.1)	1.7 (1.0)	1.7 (1.1)	6/9
Drug rash with eosinophilia and systemic symptoms syndrome	8 (9.9)	3.3 (2.4)	1.9 (1.5)	7/8
Agranulocytosis	8 (9.9)	2.1 (1.4)	2.0 (1.6)	4/8
Tubulo-interstitial nephritis	7 (8.6)	3.0 (1.5)	2.6 (1.5)	5/7
Localized skin eruption due to drugs and medicaments taken internally	6 (7.4)	1.8 (1.2)	1.3 (0.5)	4/6
Drug-induced acute pancreatitis	3 (3.7)	1.3 (0.6)	1.3 (0.6)	2/3
Acute myocarditis	2 (2.5)	2.0 (0.0)	2.0 (0.0)	0/2
Encephalitis, myelitis and encephalomyelitis	2 (2.5)	2.5 (0.7)	2.5 (0.7)	0/2
Eosinophilia	2 (2.5)	2.0 (0.0)	2.0 (0.0)	1/2
Angioneurotic oedema	1 (1.2)	1.0 (0.0)	1.0 (0.0)	1/1
Aplastic anaemia	1 (1.2)	2.0 (0.0)	0.0 (0.0)	0/1
Thrombocytopenia	1 (1.2)	2.0 (0.0)	1.0 (0.0)	1/1
Toxic erythema	1 (1.2)	2.0 (0.0)	2.0 (0.0)	1/1
Grand Total	81 (100.0)	2.2 (1.5)	1.8 (1.3)	51/81

Both drug- and case-level analyses indicate that dermatologic syndromes (including DRESS) and drug-induced liver injury concentrate higher LTT positivity, with SPS means that are consistent with likely drug causation. Tubulo-interstitial nephritis combines substantial polyexposure with frequent LTT positivity and a high number of suspects meeting SPS ≥ 4 per case. In contrast, hematologic (agranulocytosis) and neuro-cardiac phenotypes show lower or absent LTT positivity, despite multiple suspected drugs.

### Relation between SPS score and result of LTT

Overall, 77 out of 188 LTTs performed were positive (40.5%), enabling the identification of at least one culprit drug in 62.0% of the total 92 ADR episodes (57/92). Among the 66 positive LTTs assessed with SPS score, 64 (96.9%) had SPS score ≥+4 while only 2 (3.1%) had a previous SPS score <+4. On the other hand, among negative LTTs, 24.7% (24/97) had a prior SPS score <+4 and 75.3% (73/97) had a score ≥+4. Statistically significant differences were observed in the association between LTT positivity and SPS causality score ≥4 (*p* = 0.0004). This indicates that the LTT frequently identified the specific culprit within a multi-drug exposure, effectively clearing other highly suspected agents. Detailed characteristics for each case, including individual drugs causality scores and LTT results, are presented in the **Supplementary Material** (**Supplementary Table 3**).

### Drug tolerance after ADR episode

Patients with drugs that produced a positive LTT result were not deliberately re-challenged. In most instances, re-exposure occurred only when physicians, after completing a comprehensive clinical assessment, had ruled out drug allergy by considering not only the LTT findings but also results from other allergy tests, even in cases where the LTT was positive. DPT was not classified as re-exposure.

We analyzed 53 drug re-exposures conducted in 28 patients. Overall, tolerance to re-exposure was high (48/53; 90.6%). Only five re-exposures (9.4%) resulted in a recurrence of symptoms, which recapitulated the original phenotype (rash, generalized drug eruption, DRESS, and tubulo-interstitial nephritis) (**Table 6**).

**Table 6:** Cases with drug re-exposure after an ADR episode.

Case	RAM	Drug with reNDexposure	SPS score	LTT result	Prick test	Intradermal test	Patch test	Provocation test	Tolerance to drug reNDexposure
5	Rash and other nonspecific skin eruptions	Clindamycin	6	ND	Neg	Neg	ND	Neg	Yes
6	Rash and other nonspecific skin eruptions	Ethambutol	2	ND	Neg	ND	Neg	Neg	Yes
		Levofloxacin	4	Pos	Neg	ND	Neg	Pos	Yes
		Linezolid	3	Pos	Neg	ND	Neg	Pos	Yes
		Protionamide	2	Neg	Neg	ND	Neg	Neg	Yes
		Pyrazinamide	8	Neg	Neg	ND	Neg	Pos	Yes
7	Rash and other nonspecific skin eruptions	Albendazole	2	Neg	ND	ND	ND	Neg	Yes
		Levofloxacin	1	Neg	ND	ND	ND	Neg	Yes
		Sulfamethoxazole and trimethoprim	1	Neg	ND	ND	ND	Neg	Yes
12	Urticaria	Amoxicillin	6	Neg	Neg	Neg	ND	Neg	Yes
14	Drug rash with eosinophilia and systemic symptoms syndrome	Meropenem	8	Pos	ND	ND	ND	ND	No (Rash and other nonspecific skin eruptions)
15	Drug rash with eosinophilia and systemic symptoms syndrome	Amoxicillin	2	Neg	ND	ND	ND	ND	Yes
		Ibuprofen	6	Pos	ND	ND	ND	ND	Yes
		Paracetamol	1	Neg	ND	ND	ND	ND	Yes
16	Tubulo-interstitial nephritis	Mycophenolate mofetil	2	ND	ND	ND	ND	ND	No (TubuloNDinterstitial nephritis)
		Sulfamethoxazole and trimethoprim	9	Pos	ND	ND	ND	ND	No (TubuloNDinterstitial nephritis)
17	Agranulocytosis	Cefuroxime	7	Neg	ND	ND	ND	ND	Yes
		Metamizole	6	Neg	ND	ND	ND	ND	Yes
		Paracetamol	8	Neg	ND	ND	ND	ND	Yes
18	Drug rash with eosinophilia and systemic symptoms syndrome	Metamizole	-1	Neg	ND	ND	ND	ND	Yes
		Paracetamol	-2	Neg	ND	ND	ND	ND	Yes
19	Drug rash with eosinophilia and systemic symptoms syndrome	Amoxicillin/Clavulanic acid	4	Neg	ND	ND	ND	ND	Yes
		Meropenem	5	Neg	ND	ND	ND	ND	Yes
		Metamizole	2	Neg	Neg	Neg	ND	ND	Yes
19	Rash and other nonspecific skin eruptions	Baclofen	4	ND	ND	ND	ND	ND	Yes
		Cisatracurium	4	Neg	ND	ND	ND	ND	Yes
		Indomethacin	4	Pos	ND	ND	ND	ND	Yes
		Metamizole	7	Neg	Neg	Neg	ND	ND	Yes
26	Rash and other nonspecific skin eruptions	Amoxicillin/Clavulanic acid	8	Pos	ND	ND	ND	ND	No (Rash and other nonspecific skin eruptions)
31	Eosinophilia	Ondansetron	6	Pos	ND	ND	ND	ND	Yes
		Ranitidine	5	Neg	ND	ND	ND	ND	Yes
33	Rash and other nonspecific skin eruptions	Cefotaxime	5	ND	Neg	Neg	Neg	Neg	Yes
34	Rash and other nonspecific skin eruptions	Amoxicillin	6	ND	Neg	Neg	Neg	Neg	Yes
39	Generalized skin eruption due to drugs and medicaments taken internally	Amoxicillin	10	Pos	Neg	Neg	ND	Pos	No (Generalized skin eruption due to drugs and medicaments taken internally)
43	Generalized skin eruption due to drugs and medicaments taken internally	Ibuprofen	8	Pos	Neg	ND	Neg	Neg	Yes
		Paracetamol	8	Pos	Neg	Neg	Neg	Neg	Yes
44	Localized skin eruption due to drugs and medicaments taken internally	Paracetamol	6	Neg	Neg	Neg	ND	Neg	Yes
47	Agranulocytosis	Paracetamol	3	Neg	ND	ND	ND	ND	Yes
49	Generalized skin eruption due to drugs and medicaments taken internally	Ibuprofen	6	Neg	ND	ND	Neg	Neg	Yes
		Paracetamol	6	Neg	ND	ND	Neg	Neg	Yes
52	Urticaria	Amoxicillin	7	Neg	ND	ND	ND	ND	Yes
53	Localized skin eruption due to drugs and medicaments taken internally	Amoxicillin/Clavulanic acid	6	Neg	ND	ND	ND	Neg	Yes
60	Encephalitis, myelitis and encephalomyelitis	PEG 2000	4	Neg	ND	ND	ND	ND	Yes
		PS 80	4	Neg	ND	ND	ND	ND	Yes
61	Aplastic anaemia	PEG 2000	3	Neg	ND	ND	ND	ND	Yes
		PS 80	3	Neg	ND	ND	ND	ND	Yes
66	Rash and other nonspecific skin eruptions	Amoxicillin	5	Pos	ND	ND	ND	ND	Yes
72	Generalized skin eruption due to drugs and medicaments taken internally	Amoxicillin	3	ND	ND	ND	ND	Neg	Yes
		Dexchlorpheniramine	4	Pos	Neg	ND	ND	ND	Yes
		Ibuprofen	4	Neg	Neg	Neg	ND	Neg	Yes
78	Agranulocytosis	Mycophenolate mofetil	5	Neg	ND	ND	ND	ND	Yes
79	Generalized skin eruption due to drugs and medicaments taken internally	Amoxicillin	4	Neg	Neg	Neg	Neg	Neg	Yes
80	Urticaria	Paracetamol	5	ND	Neg	Neg	ND	Neg	Yes

ND, Not Done.

The agents most often re-introduced were amoxicillin (n=8; 7 tolerated, 1 not), paracetamol (n=8; 8 tolerated), ibuprofen (n=4; 4 tolerated), and metamizole (n=4; 4 tolerated). Among excipients, PEG 2000 (n=2) and polysorbate 80 (n=2) were re-exposed without incident, each with negative LTT.

In this real-world series, re-exposure after ADR was generally safe, with >90% tolerance across 53 challenges. LTT negativity was a strong predictor of successful re-exposure; conversely, LTT positivity identified a subgroup at higher risk of failure, though most LTT-positive challenges still proceeded uneventfully. Provocation testing added limited incremental predictive value. These findings support a risk-stratified approach to re-exposure, considering re-challenge when LTT is negative, with close monitoring.

### Diagnostic accuracy and concordance of testing modalities

To assess diagnostic accuracy, an *ad-hoc* analysis was performed using drug tolerance upon re-exposure as the reference standard. The resulting sensitivity, specificity, PPV, and NPV for each test are summarized in **Table 7**.

**Table 7:** Test characteristics.

Test	Sensitivity (%), IC 95%	Specificity (%), IC 95%	Positive predictive value (PPV) (%), IC 95%	Negative predictive value (NPV) (%), IC 95%
LTT	100 (51.0-100)	78.0 (63.3-88.0)	30.8 (12.6-57.6)	100 (89.3 – 100)
SPS score≥4	75 (36.5-94.0)	31.6 (20.5-45.6)	11.8 (25.8-49.5)	91.2 (69.2-97.9)
Skin Prick-test (SPT)	25 (2.7-80.2)	97.4 (79.1-99.8)	50 (5.5-94.5)	92.5 (73.1-98.2)
Patch test	50 (5.5-94.5)	96.2 (71.7-99.6)	50 (5.5-94.5)	96.2 (71.7-99.6)
Intradermal test (IDT)	25 (2.7-80.2)	95.8 (69.9-99.6)	50 (5.5-94.5)	88.5 (62.1-97.3)
Drug provocation test (DPT)	75 (19.8-97.3)	84.8 (65.3-94.3)	30 (7.3-70.1)	97.5 (80.0-99.7)

Among the tested modalities, the LTT demonstrated perfect sensitivity (100%) and NPV (100%), though its specificity was moderate (78%), which contributed to a lower PPV (30.8%). In contrast, all skin tests (prick, patch, and intradermal) were highly specific (96–97%) but exhibited low sensitivity (25–50%), resulting in modest PPVs and high NPVs. While using an SPS score of ≥4 increased sensitivity to 75%, it did so at the expense of very low specificity (32%). The DPT provided the most balanced diagnostic profile, with a sensitivity of 75% and a specificity of 85%, NPV 97.5%.

Overall, these results indicate that most modalities were more reliable for ruling out the diagnosis (high NPV) than for confirming it (generally low PPV). Statistically significant differences were observed in the association between LTT positivity and SPS causality score ≥4 (p < 0.001), between results of LTT and DPT (p-value = 0.008). There were no statistically significant differences among other types of skin tests (SPT, patch testing, IDT) and LTT. This latter finding is likely due to the study’s limited statistical power.

## Discussion

Reliable epidemiological data on ADRs are generally scarce, particularly in the pediatric population (7). The role of age as a potential risk factor for ADRs remains controversial: while some studies suggest that younger children are at greater risk, others report the opposite (35, 36). In our cohort, two peaks of ADR occurrence were identified, at ages 0–5 and 14–18 years, with a mean age of 9.5 ± 5.9 years.

A slight predominance of ADRs was observed in males (53.1%). This male predominance has similarly been reported in Spain (37), in a Canadian hospital (38) and in analyses based on VigiBase (7). By contrast, in adult populations, ADRs are more frequently reported among females (39).

Non-steroidal anti-inflammatory drugs (NSAIDs) and antibiotics consistently emerge as the drug classes most frequently associated with drug hypersensitivity reactions. Some studies have identified antibiotics as the leading cause of ADRs in young children, whereas others indicate that NSAIDs predominate in both children and adolescents, particularly in severe cases (9, 40).

Our epidemiological findings are consistent with this evidence: β-lactam antibiotics accounted for the highest proportion of implicated drugs (28%), with non-steroidal analgesic drugs also prevalent (14%). These elevated frequencies likely reflect prescription patterns in pediatrics, where antibiotics are widely used, and NSAIDs are routinely prescribed for the management of pain and fever.

ADRs present a wide clinical spectrum. Classically, urticaria and angioedema are indicative of immediate-type hypersensitivity, whereas maculopapular exanthems are more consistent with delayed-type reactions (12, 41). In our cohort, cutaneous involvement was frequent (70%), with maculopapular rash as the predominant manifestation (41%).

Because infections, particularly viral, are highly prevalent in childhood, they constitute both important risk factors and key differential diagnoses for drug hypersensitivity (12). Underlying viral pathogens may also act as cofactors in susceptible individuals (42). In this regard, aminopenicillins are well recognized to provoke exanthems in the context of Epstein–Barr virus (EBV) infection (12, 42), concordantly, two patients in our series with confirmed EBV developed an exanthematous eruption. Likewise, human herpesvirus 6 (HHV-6) reactivation has been implicated as a cofactor in drug reaction with eosinophilia and systemic symptoms (DRESS) (12). In our study, HHV-6 was detected in 3/8 DRESS cases (37,5%), supporting a contributory role in a substantial subset.

The most frequently administered therapies for ADRs were dexchlorpheniramine (24.0%), methylprednisolone (14.9%), and prednisone (7.4%). These agents represent standard management for suspected drug hypersensitivity with cutaneous involvement (43).

Establishing drug hypersensitivity diagnostics in pediatric patients requires a comprehensive allergy assessment: clinical history supplemented by available *in vivo* and *in vitro* tests. Most pediatric evaluations of skin tests performance pertain to antibiotics (17, 44–47). For immediate reactions (≤1 hour after drug exposure), particularly to β-lactams, SPT and IDT demonstrate comparatively good diagnostic utility; by contrast, recent pediatric studies report low sensitivity of these tests for non-immediate hypersensitivity (14–19). Substantial evidence indicates that delayed drug hypersensitivity is predominantly T-cell–mediated; consequently, SPT and IDT, which assess IgE-mediated allergy mechanisms, are suboptimal for establishing the diagnosis in this context (48, 49). Patch test could be useful for delayed hypersensitivity, especially to anticonvulsants and NSAIDs; however, larger studies are required to substantiate its diagnostic performance (50, 51). In our *ad-hoc* analysis using drug re-challenge as the reference standard, the sensitivities were 25% for both SPT and IDT, and 50% for patch testing, that correlates with previous literature findings.

DPT, also termed drug challenge, is a key diagnostic tool in the evaluation of suspected drug allergy in both adults and children. In pediatrics, DPT is regarded as the diagnostic gold standard for drug hypersensitivity (21, 30), demonstrating high sensitivity and specificity, with an estimated negative predictive value (NPV) of approximately 96.6% (17, 52, 53). The DPT in our study offered 75% sensitivity, 85% specificity and NPV of 97.5%, consistent with previously reported findings.

Evidence on the LTT in pediatric non-immediate drug hypersensitivity reactions remains limited, with most studies focusing on antibiotics and anticonvulsants (54, 55). Recent data for mild β-lactam reactions show sensitivity of 52%, specificity of 92%, PPV 86%, NPV 65% (46). In a cohort of 17 children, LTT with amoxicillin reached sensitivity of 63.6%, specificity of 100%, PPV 100%, NPV 60%, whereas LTT with clavulanate had lower sensitivity of 33.3% with identical specificity and predictive values (56).

In our cohort, among 66 positive LTTs antibacterials (ATC J01) comprised 62.1% (41/66), followed by antiepileptics at 10.6% (7/66) and analgesics (N02) at 6.1% (4/66). Our study confirms that LTT positivity in this cohort was predominantly driven by drug classes frequently associated with hypersensitivity, such as antibacterials and antiepileptics. This finding is consistent with existing literature and reinforces the test’s relevance for common clinical scenarios (9, 40, 54, 55).

When stratified by ADR phenotype, LTT positivity clustered in cutaneous syndromes (generalized eruptions, DRESS, localized eruptions, nonspecific rash) comprising 63.6% (49/77) and in drug-induced liver injury,14.3% (11/77). We did not stratify LTT sensitivity, specificity, PPV, or NPV by drug class or ADR phenotype because of the limited sample size. The concentration of positive results in specific phenotypes like severe cutaneous syndromes and drug-induced liver injury highlights the LTT’s utility in diagnosing T-cell mediated reactions, which are the immunological basis for these conditions (12, 41, 48, 49).

The LTT’s diagnostic performance was a standout finding of our study. Its perfect sensitivity and NPV (100%) establish it as a powerful rule-out tool, allowing clinicians to confidently clear a suspected drug based on a negative result. Although its moderate specificity (78%) necessitates careful interpretation of positive results, the test’s clinical validity is reinforced by its strong correlation with high SPS causality scores (≥4) (p < 0.001). When contextualized against other diagnostics, the LTT’s role becomes even clearer. The test showed significant concordance with the DPT (p < 0.01). In contrast, while no statistically significant difference was found when comparing it to skin tests, this is likely a result of the study’s limited statistical power, as their clinical profiles are near-opposites. This ultimately positions the LTT as a crucial, non-invasive instrument within the diagnostic algorithm. It functions as a robust rule-out tool, complementing the weak rule-in capacity of skin tests and serving as a safe alternative when the DPT - the definitive final arbiter - is considered too risky.

A recent meta-analysis indicates that the optimal window to perform the lymphocyte transformation test (LTT) is 2 weeks to 2 months after the initial clinical manifestation; the poorest performance is observed within the first 2 weeks and beyond 2 months, with an additional decline after approximately 36 months (57). In our cohort, the median interval to LTT was 176 [117-324] days. This wide dispersion reflects that some patients reported ADR manifestations years earlier that were not investigated at that time, and these historical episodes were documented as separate events. Excluding four extreme historical outliers (1054; 1807; 2449; 6353 days), the interquartile range decreases to 112-304.8 days (~ 4-10 months), a timeframe consistent with acceptable LTT performance and thus supportive of the reliability of our results.

Despite the frequency of reported allergies, avoidance of penicillin is not necessary in most individuals. After appropriate allergy evaluation, approximately 90–95% of labeled patients can tolerate β-lactam re-exposure (58). Our data are directionally consistent with these estimates: among patients re-challenged with penicillins, 13 of 15 (86%) tolerated re-exposure without recurrence, underscoring the value of systematic assessment and delabeling to optimize antimicrobial stewardship.

### Strengths and limitations

The key strengths of this study include a real-world pediatric cohort with diverse phenotypes, use of a clinically meaningful reference standard (tolerance on re-exposure) to derive diagnostic accuracy, and direct head-to-head comparisons across diagnostic modalities. Limitations comprise the retrospective design with selection bias, including who underwent *in-vitro* testing and who was subsequently re-exposed; only 28 patients contributed 53 re-exposures, which constrains precision of accuracy estimates. Although causality scoring was applied retrospectively for some cases and explicitly without incorporating laboratory, allergy testing, or re-exposure outcomes, residual risk of information bias remains.

Regarding generalizability, our findings reflect the clinical reality of a tertiary care hospital, which may limit the external validity to primary care settings or less complex facilities. However, the consistency of our epidemiological data with international registries suggests that our results concerning LTT diagnostic accuracy are applicable to similar pediatric populations undergoing specialized allergy evaluation.

## Conclusions

In our pediatric cohort, LTT positivity correlated significantly with high SPS causality scores (≥4) and showed a predictable discordance with DPT outcomes. These findings reinforce the LTT’s clinical utility as a valuable, non-invasive tool for evaluating suspected T-cell–mediated reactions, especially when a DPT is contraindicated or considered too risky.

## Data Availability

The raw data supporting the conclusions of this article will be made available by the authors, without undue reservation.
