# Supplementary material for: Lymphocyte transformation test in pediatric drug hypersensitivity: case series report from a tertiary care hospital
Source: Front Immunol. 2026 May 15;17:1787795. doi: 10.3389/fimmu.2026.1787795 (PMC13219294; doi:10.3389/fimmu.2026.1787795)
Supplement: Supplementary file 1 [file Table1.docx]

**Supplementary material**

**TABLE S1. Tests performed**

| Blood test, n (%) | | |  |
| --- | --- | --- | --- |
| - Negative | | | 23 (25.0) |
| - Positive | | | 54 (58.7) |
| - Not performed | | | 15 (16.3) |
| Microbiology tests, n (%) | | | |
| - Negative | | | 47 (51.1) |
| - Positive | | | 26 (28.2) |
| - Not performed | | | 19 (20.7) |
| By type of microbiology test | | | |
| - - Serology | | | |
| Number of positive tests (n (%)) | Result | Type of ADR | Number of positive tests (n (%)) |
| 13 (72.2) | HHV 6 | Drug-induced liver injury | 3 (23.1) |
|  |  | DRESS | 3 (23.1) |
|  |  | Urticaria | 2 (15.4) |
|  |  | Tubulo-interstitial nephritis | 2 (15.4) |
|  |  | Rash and other nonspecific skin eruptions | 1 (7.7) |
|  |  | Localized skin eruption due to drugs and medicaments taken internally | 1 (7.7) |
|  |  | Generalized skin eruption due to drugs and medicaments taken internally | 1 (7.7) |
| 3 (16.7) | Mycoplasma IgM | Tubulo-interstitial nephritis | 1 (33.3) |
|  |  | Rash and other nonspecific skin eruptions | 2 (66.6) |
| 2 (11.1) | EBV IgM | Rash and other nonspecific skin eruptions | 1 (50.0) |
|  |  | DRESS | 1 (50.0) |
| 1 (5.6) | Parotitis IgM | Drug-induced liver injury | 1 (100.0) |
| 1 (5.6) | SARS-COV2 | Eosinophilia | 1 (100.0) |
| - - Blood culture, number of positive tests (n (%)) | | | |
| 1 (50.0) | Staphylococcus aureus | Rash and other nonspecific skin eruptions | |
| 1 (50.0) | Streptococcus salivarius | Agranulocytosis | |
| - - Urine culture, number of positive tests (n (%)) | | | |
| 1 (50.0) | Escherichia coli | Generalized skin eruption due to drugs and medicaments taken internally | |
| 1 (50.0) | Klebsiella pneumoniae | Acute pancreatitis | |
| - - PCR, number of positive tests (n (%)) | | | |
| 1 (25.0) | EBV, BK virus | Tubulo-interstitial nephritis | |
| 1 (25.0) | COVID-19 | Tubulo-interstitial nephritis | |
| 2 (50.0) | HHV I | Tubulo-interstitial nephritis | |
| - - Sputum study, number of positive tests (n (%)) | | | |
| 1 (100.0) | Mycobacterium tuberculosis complex | Drug-induced liver injury | |
| - - CSF, study, number of positive tests (n (%)) | | | |
| 1 (100.0) | Staphylococcus aureus | Agranulocytosis | |
| *Abbreviations: ADRs, adverse drug reaction; IQR, interquartile range;* *PCR, Polymerase Chain Reaction; HHV-6, Human herpesvirus 6; EBV, Epstein-Barr Virus;* *CSF, cerebrospinal fluid.* | | | |

| **TABLE S2. Characteristics of culprit drugs** |  |
| --- | --- |
| Number of drugs studied per ADR episode, n (%) |  |
| - One | 38 (41.3) |
| - Two | 30 (32.6) |
| - Three | 9 (9.8) |
| - Four | 5 (5.4) |
| - Five | 4 (4.3) |
| - Six | 5 (5.4) |
| - Seven | 1 (1.1) |
| Number of drugs studied per ADR episode (mean (SD)) | 2.2 (1.5) |
| Drugs studied, n (%) | 202 (100.0) |
| - Amoxicillin | 17 (8.4) |
| - Paracetamol | 16 (7.9) |
| - Amoxicillin/clavulanic acid | 14 (6.9) |
| - Ibuprofen | 13 (6.4) |
| - Other antiviral vaccines | 12 (5.9) |
| - Metamizole | 10 (5.0) |
| - Cefotaxime | 7 (3.5) |
| - Meropenem | 7 (3.5) |
| - Sulfamethoxazole and Trimethoprim | 6 (3.0) |
| - Levetiracetam | 5 (2.5) |
| - Clindamycin | 4 (2.0) |
| - Fluconazole | 4 (2.0) |
| - Metronidazole | 4 (2.0) |
| - Piperacillin + tazobactam | 3 (1.5) |
| - Cefuroxime | 3 (1.5) |
| - Levofloxacin | 3 (1.5) |
| - Vancomycin | 3 (1.5) |
| - Rifampicin | 3 (1.5) |
| - Pyrazinamide | 3 (1.5) |
| - Naproxen | 3 (1.5) |
| - Ranitidine | 2 (1.0) |
| - Omeprazole | 2 (1.0) |
| - Ceftriaxone | 2 (1.0) |
| - Azithromycin | 2 (1.0) |
| - Teicoplanin | 2 (1.0) |
| - Isoniazid | 2 (1.0) |
| - Ethambutol | 2 (1.0) |
| - Mycophenolate mofetil | 2 (1.0) |
| - Dexketoprofen | 2 (1.0) |
| - Tramadol | 2 (1.0) |
| - Topiramate | 2 (1.0) |
| - Esomeprazole | 1 (0.5) |
| - Ondansetron | 1 (0.5) |
| - Macrogol | 1 (0.5) |
| - Mesalazine | 1 (0.5) |
| - Indomethacin | 1 (0.5) |
| - Omega-3 Triglycerides, Including Other Esters and Acids | 1 (0.5) |
| - Tacrolimus | 1 (0.5) |
| - Tetracycline | 1 (0.5) |
| - Ampicillin | 1 (0.5) |
| - Phenoxymethylpenicillin | 1 (0.5) |
| - Cefixime | 1 (0.5) |
| - Cefepime | 1 (0.5) |
| - Trimethoprim | 1 (0.5) |
| - Sulfamethoxazole | 1 (0.5) |
| - Erythromycin | 1 (0.5) |
| - Fosfomycin | 1 (0.5) |
| - Linezolid | 1 (0.5) |
| - Micafungin | 1 (0.5) |
| - Cycloserine | 1 (0.5) |
| - Protionamide | 1 (0.5) |
| - Ganciclovir | 1 (0.5) |
| - Valganciclovir | 1 (0.5) |
| - Sirolimus | 1 (0.5) |
| - Rocuronium bromide | 1 (0.5) |
| - Cisatracurium | 1 (0.5) |
| - Baclofen | 1 (0.5) |
| - Fentanyl | 1 (0.5) |
| - Propofol | 1 (0.5) |
| - Oxcarbazepine | 1 (0.5) |
| - Eslicarbazepine | 1 (0.5) |
| - Valproic acid | 1 (0.5) |
| - Diazepam | 1 (0.5) |
| - Albendazole | 1 (0.5) |
| - Guaifenesin | 1 (0.5) |
| - Guaifenesin + pseudoephedrine | 1 (0.5) |
| - Dextromethorphan | 1 (0.5) |
| - Dexchlorpheniramine | 1 (0.5) |
| - Acetazolamide | 1 (0.5) |
| - Ciprofloxacin | 1 (0.5) |
| - Rasburicase | 1 (0.5) |
| Drugs studied per ADR type, n(%) | |
| 1. Rash and other nonspecific skin eruptions | 43 (100.0) |
| - 1. Amoxicillin | 4 (9.3) |
| - 1. Cefotaxime | 3 (7.0) |
| - 1. Clindamycin | 3 (7.0) |
| - 1. Metamizole | 3 (7.0) |
| - 1. Amoxicillin/Clavulanic acid | 2 (4.7) |
| - 1. Meropenem | 2 (4.7) |
| - 1. Sulfamethoxazole and trimethoprim | 2 (4.7) |
| - 1. Levofloxacin | 2 (4.7) |
| - 1. Paracetamol | 2 (4.7) |
| - 1. Indomethacin | 1 (2.3) |
| - 1. Ampicillin | 1 (2.3) |
| - 1. Trimethoprim | 1 (2.3) |
| - 1. Sulfamethoxazole | 1 (2.3) |
| - 1. Teicoplanin | 1 (2.3) |
| - 1. Linezolid | 1 (2.3) |
| - 1. Micafungin | 1 (2.3) |
| - 1. Cycloserine | 1 (2.3) |
| - 1. Protionamide | 1 (2.3) |
| - 1. Pyrazinamide | 1 (2.3) |
| - 1. Ethambutol | 1 (2.3) |
| - 1. Ganciclovir | 1 (2.3) |
| - 1. Ibuprofen | 1 (2.3) |
| - 1. Rocuronium bromide | 1 (2.3) |
| - 1. Cisatracurium | 1 (2.3) |
| - 1. Baclofen | 1 (2.3) |
| - 1. Fentanyl | 1 (2.3) |
| - 1. Oxcarbazepine | 1 (2.3) |
| - 1. Eslicarbazepine | 1 (2.3) |
| - 1. Albendazole | 1 (2.3) |
| 1. Urticaria | 18 (100.0) |
| - 1. Amoxicillin | 4 (22.2) |
| - 1. Amoxicillin/Clavulanic acid | 3 (16.7) |
| - 1. Dexketoprofen | 2 (11.1) |
| - 1. Cefuroxime | 1 (5.6) |
| - 1. Cefotaxime | 1 (5.6) |
| - 1. Metamizole | 1 (5.6) |
| - 1. Sulfamethoxazole and trimethoprim | 1 (5.6) |
| - 1. Levofloxacin | 1 (5.6) |
| - 1. Rifampicin | 1 (5.6) |
| - 1. Other antiviral vaccines | 1 (5.6) |
| - 1. Tramadol | 1 (5.6) |
| - 1. Paracetamol | 1 (5.6) |
| 1. Drug-induced liver injury | 27 (100.0) |
| - 1. Paracetamol | 3 (11.1) |
| - 1. Other antiviral vaccines | 3 (11.1) |
| - 1. Ceftriaxone | 2 (7.4) |
| - 1. Isoniazid | 2 (7.4) |
| - 1. Pyrazinamide | 2 (7.4) |
| - 1. Ethambutol | 2 (7.4) |
| - 1. Amoxicillin/Clavulanic acid | 2 (7.4) |
| - 1. Azithromycin | 1 (3.7) |
| - 1. Fluconazole | 1 (3.7) |
| - 1. Ranitidine | 1 (3.7) |
| - 1. Rifampicin | 1 (3.7) |
| - 1. Guaifenesin | 1 (3.7) |
| - 1. Dextromethorphan | 1 (3.7) |
| - 1. Ibuprofen | 1 (3.7) |
| - 1. Piperacillin + tazobactam | 1 (3.7) |
| - 1. Erythromycin | 1 (3.7) |
| - 1. Propofol | 1 (3.7) |
| - 1. Levetiracetam | 1 (3.7) |
| 1. Generalized skin eruption due to drugs and medicaments taken internally | 15 (100.0) |
| - 1. Amoxicillin | 5 (33.3) |
| - 1. Paracetamol | 3 (20.0) |
| - 1. Tacrolimus | 2 (13.3) |
| - 1. Mycophenolate mofetil | 1 (6.7) |
| - 1. Guaifenesin + pseudoephedrine | 1 (6.7) |
| - 1. Clindamycin | 1 (6.7) |
| - 1. Naproxen | 1 (6.7) |
| - 1. Dexchlorpheniramine | 1 (6.7) |
| 1. Drug rash with eosinophilia and systemic symptoms syndrome | 26 (100.0) |
| - 1. Amoxicillin/Clavulanic acid | 4 (15.4) |
| - 1. Meropenem | 4 (15.4) |
| - 1. Amoxicillin | 2 (7.7) |
| - 1. Piperacillin + tazobactam | 2 (7.7) |
| - 1. Cefotaxime | 2 (7.7) |
| - 1. Vancomycin | 2 (7.7) |
| - 1. Metronidazole | 2 (7.7) |
| - 1. Metamizole | 2 (7.7) |
| - 1. Paracetamol | 2 (7.7) |
| - 1. Levetiracetam | 2 (7.7) |
| - 1. Fluconazole | 1 (3.8) |
| 1. Agranulocytosis | 17 (100.0) |
| - 1. Paracetamol | 2 (11.8) |
| - 1. Metamizole | 2 (11,8) |
| - 1. Omeprazole | 1 (5.9) |
| - 1. Amoxicillin | 1 (5.9) |
| - 1. Cefuroxime | 1 (5.9) |
| - 1. Cefotaxime | 1 (5.9) |
| - 1. Cefixime | 1 (5.9) |
| - 1. Cefepime | 1 (5.9) |
| - 1. Vancomycin | 1 (5.9) |
| - 1. Teicoplanin | 1 (5.9) |
| - 1. Metronidazole | 1 (5.9) |
| - 1. Valganciclovir | 1 (5.9) |
| - 1. Mycophenolate mofetil | 1 (5.9) |
| - 1. Ibuprofen | 1 (5.9) |
| - 1. Acetazolamide | 1 (5.9) |
| 1. Tubulo-interstitial nephritis | 21 (100.0) |
| - 1. Ibuprofen | 3 (14.3) |
| - 1. Sulfamethoxazole and trimethoprim | 2 (9.5) |
| - 1. Omeprazole | 1 (4.8) |
| - 1. Esomeprazole | 1 (4.8) |
| - 1. Omega-3 triglycerides, including other esters and acids | 1 (4.8) |
| - 1. Tacrolimus | 1 (4.8) |
| - 1. Amoxicillin | 1 (4.8) |
| - 1. Phenoxymethylpenicillin | 1 (4.8) |
| - 1. Amoxicillin/Clavulanic acid | 1 (4.8) |
| - 1. Cefuroxime | 1 (4.8) |
| - 1. Fosfomycin | 1 (4.8) |
| - 1. Sirolimus | 1 (4.8) |
| - 1. Mycophenolate mofetil | 1 (4.8) |
| - 1. Colchicina | 1 (4.8) |
| - 1. Metamizole | 1 (4.8) |
| - 1. Paracetamol | 1 (4.8) |
| - 1. Ciprofloxacin | 1 (4.8) |
| - 1. Rasburicasa | 1 (4.8) |
| 1. Localized skin eruption due to drugs and medicaments taken internally | 11 (100.0) |
| - 1. Amoxicillin/Clavulanic acid | 2 (18.2) |
| - 1. Paracetamol | 2 (18.2) |
| - 1. Topiramate | 2 (18.2) |
| - 1. Levetiracetam | 2 (18.2) |
| - 1. Ibuprofen | 1 (9.1) |
| - 1. Sulfamethoxazole and Trimethoprim | 1 (9.1) |
| - 1. Metamizole | 1 (9.1) |
| 1. Drug-induced acute pancreatitis | 4 (100.0) |
| - 1. Mesalazine | 1 (25.0) |
| - 1. Naproxen | 1 (25.0) |
| - 1. Ibuprofen | 1 (25.0) |
| - 1. Valproic acid | 1 (25.0) |
| 1. Eosinophilia | 4 (100.0) |
| - 1. Ranitidine | 1 (25.0) |
| - 1. Ondansetron | 1 (25.0) |
| - 1. Fluconazole | 1 (25.0) |
| - 1. Meropenem | 1 (25.0) |
| 1. Encephalitis, myelitis and encephalomyelitis | 5 (100.0) |
| - 1. Other antiviral vaccines | 2 (40.0) |
| - 1. Naproxen | 1 (20.0) |
| - 1. Tramadol | 1 (20.0) |
| - 1. Diazepam | 1 (20.0) |
| 1. Acute myocarditis | 4 (100.0) |
| - 1. Other antiviral vaccines | 4 (100.0) |
| 1. Toxic erythema | 2 (100.0) |
| - 1. Metronidazole | 1 (50.0) |
| - 1. Tetracycline | 1 (50.0) |
| 1. Thrombocytopenia | 2 (100.0) |
| - 1. Fluconazole | 1 (50.0) |
| - 1. Azithromycin | 1 (50.0) |
| 1. Aplastic anaemia | 2 (100.0) |
| - 1. Other antiviral vaccines | 2 (100.0) |
| 1. Angioneurotic oedema | 1 (100.0) |
| - 1. Naproxen | 1 (100.0) |

**TABLE S3. Characteristics of cases**

| **Case** | **ADR** | **Drugs** | **Score** | | **LTT interp** | **No. of drugs assessed** | **No. of positive LTTs** |
| --- | --- | --- | --- | --- | --- | --- | --- |
|  |  |  | **SPS** | **RUCAM** |  |  |  |
| 1 | Urticaria | Cefotaxime | 7 |  | Pos | 3 | 2 |
|  |  | Metamizole | 7 |  | Neg |  |  |
|  |  | Amoxicillin/Clavulanic acid | 6 |  | Pos |  |  |
| 2 | Drug rash with eosinophilia and systemic symptoms syndrome | Cefotaxime | 7 |  | Pos | 5 | 4 |
|  |  | Levetiracetam | 7 |  | Pos |  |  |
|  |  | Vancomycin | 7 |  | Pos |  |  |
|  |  | Metronidazole | 5 |  | Neg |  |  |
|  |  | Meropenem | 6 |  | Pos |  |  |
| 3 | Generalized skin eruption due to drugs and medicaments taken internally | Ibuprofen | 5 |  | Neg | 2 | 0 |
|  |  | Guaifenesin + pseudoephedrine | 3 |  | Neg |  |  |
| 4 | Urticaria | Amoxicillin | 7 |  | Neg | 2 | 0 |
|  |  | Dexketoprofen | 7 |  | ND |  |  |
| 5 | Rash and other nonspecific skin eruptions | Cefotaxime | 7 |  | Neg | 2 | 0 |
|  |  | Clindamycin | 6 |  | ND |  |  |
| 6 | Rash and other nonspecific skin eruptions | Pyrazinamide | 8 |  | Neg | 6 | 3 |
|  |  | Protionamide | 2 |  | Neg |  |  |
|  |  | Cycloserine | 7 |  | Pos |  |  |
|  |  | Ethambutol | 2 |  | Neg |  |  |
|  |  | Levofloxacin | 4 |  | Pos |  |  |
|  |  | Linezolid | 3 |  | Pos |  |  |
| 7 | Rash and other nonspecific skin eruptions | Ampicillin | 7 |  | Pos | 6 | 1 |
|  |  | Meropenem | 7 |  | Neg |  |  |
|  |  | Sulfamethoxazole and trimethoprim | 1 |  | Neg |  |  |
|  |  | Levofloxacin | 1 |  | Neg |  |  |
|  |  | Albendazole | 2 |  | Neg |  |  |
|  |  | Metamizole | 7 |  | Neg |  |  |
| 8 | Urticaria | Amoxicillin | 8 |  | Pos | 1 | 1 |
| 9 | Rash and other nonspecific skin eruptions | Amoxicillin | 7 |  | Pos | 1 | 1 |
| 10 | Urticaria | Amoxicillin/Clavulanic acid | 7 |  | Pos | 2 | 1 |
|  |  | Cefuroxime | 6 |  | Neg |  |  |
| 11 | Drug-induced liver injury | Pyrazinamide |  | 6 | Pos | 4 | 1 |
|  |  | Isoniazid |  | 3 | Neg |  |  |
|  |  | Rifampicin |  | 3 | Neg |  |  |
|  |  | Ethambutol |  | 2 | Neg |  |  |
| 12 | Urticaria | Amoxicillin | 6 |  | Neg | 1 | 0 |
| 13 | Drug rash with eosinophilia and systemic symptoms syndrome | Amoxicillin | 4 |  | Pos | 1 | 1 |
| 14 | Drug rash with eosinophilia and systemic symptoms syndrome | Meropenem | 8 |  | Pos | 2 | 1 |
|  |  | Piperacillin + tazobactam | 1 |  | Neg |  |  |
| 15 | Drug rash with eosinophilia and systemic symptoms syndrome | Ibuprofen | 6 |  | Pos | 3 | 1 |
|  |  | Amoxicillin | 2 |  | Neg |  |  |
|  |  | Paracetamol | 1 |  | Neg |  |  |
| 16 | Tubulo-interstitial nephritis | Sulfamethoxazole and trimethoprim | 9 |  | Pos | 3 | 1 |
|  |  | Mycophenolate mofetil | 2 |  | ND |  |  |
|  |  | Rasburicasa | 1 |  | ND |  |  |
| 17 | Agranulocytosis | Metronidazole | 6 |  | Pos | 5 | 1 |
|  |  | Paracetamol | 8 |  | Neg |  |  |
|  |  | Cefuroxime | 7 |  | Neg |  |  |
|  |  | Metamizole | 6 |  | Neg |  |  |
|  |  | Cefepime | 6 |  | Neg |  |  |
| 18 | Drug rash with eosinophilia and systemic symptoms syndrome | Amoxicillin/Clavulanic acid | 4 |  | Neg | 7 | 2 |
|  |  | Meropenem | 5 |  | Pos |  |  |
|  |  | Cefotaxime | 3 |  | Neg |  |  |
|  |  | Vancomycin | -2 |  | Neg |  |  |
|  |  | Metronidazole | -2 |  | Pos |  |  |
|  |  | Metamizole | -1 |  | Neg |  |  |
|  |  | Paracetamol | -2 |  | Neg |  |  |
| 19 | Drug rash with eosinophilia and systemic symptoms syndrome | Metamizole | 2 |  | Neg | 6 | 2 |
|  |  | Amoxicillin/Clavulanic acid | 4 |  | Neg |  |  |
|  |  | Levetiracetam | 4 |  | Pos |  |  |
|  |  | Meropenem | 5 |  | Neg |  |  |
|  |  | Fluconazole | 5 |  | Neg |  |  |
|  |  | Piperacillin + tazobactam | 4 |  | Pos |  |  |
| 19 | Rash and other nonspecific skin eruptions | Rocuronium bromide | 7 |  | Pos | 2 | 1 |
|  |  | Metamizole | 7 |  | Neg |  |  |
| 19 | Rash and other nonspecific skin eruptions | Metamizole | 7 |  | Neg | 6 | 2 |
|  |  | Amoxicillin/Clavulanic acid | 7 |  | Neg |  |  |
|  |  | Fentanyl | 7 |  | Pos |  |  |
|  |  | Indomethacin | 4 |  | Pos |  |  |
|  |  | Cisatracurium | 4 |  | Neg |  |  |
|  |  | Baclofen | 4 |  | ND |  |  |
| 20 | Tubulo-interstitial nephritis | Amoxicillin | 6 |  | Pos | 3 | 1 |
|  |  | Esomeprazole | 5 |  | Neg |  |  |
|  |  | Ibuprofen | 5 |  | Neg |  |  |
| 21 | Rash and other nonspecific skin eruptions | Ibuprofen | 2 |  | Neg | 2 | 0 |
|  |  | Paracetamol | 2 |  | Neg |  |  |
| 22 | Tubulo-interstitial nephritis | Sirolimus | 4 |  | Pos | 5 | 2 |
|  |  | Tacrolimus | 4 |  | Pos |  |  |
|  |  | Omeprazole | 5 |  | Neg |  |  |
|  |  | Omega-3 triglycerides, including other esters and acids | 3 |  | Neg |  |  |
|  |  | Sulfamethoxazole and Trimethoprim | 5 |  | Neg |  |  |
| 23 | Drug-induced liver injury | Pyrazinamide |  | 5 | Neg | 3 | 0 |
|  |  | Isoniazid |  | 5 | Neg |  |  |
|  |  | Rifampicin |  | 9 | Neg |  |  |
| 24 | Tubulo-interstitial nephritis | Amoxicillin/Clavulanic acid | 7 |  | Neg | 4 | 1 |
|  |  | Ibuprofen | 7 |  | Pos |  |  |
|  |  | Fosfomycin | 5 |  | Neg |  |  |
|  |  | Phenoxymethylpenicillin | 7 |  | Neg |  |  |
| 25 | Toxic erythema | Tetracycline | 7 |  | Pos | 2 | 1 |
|  |  | Metronidazole | 7 |  | Neg |  |  |
| 26 | Rash and other nonspecific skin eruptions | Amoxicillin/Clavulanic acid | 8 |  | Pos | 1 | 1 |
| 27 | Rash and other nonspecific skin eruptions | Eslicarbazepine | 7 |  | Neg | 1 | 0 |
| 28 | Drug-induced liver injury | Ceftriaxone |  | 4 | Neg | 1 | 0 |
| 29 | Drug rash with eosinophilia and systemic symptoms syndrome | Amoxicillin/Clavulanic acid | 11 |  | Pos | 1 | 1 |
| 30 | Rash and other nonspecific skin eruptions | Amoxicillin | 5 |  | Pos | 1 | 1 |
| 31 | Eosinophilia | Ondansetron | 6 |  | Pos | 2 | 1 |
|  |  | Ranitidine | 5 |  | Neg |  |  |
| 32 | Drug-induced liver injury | Amoxicillin/Clavulanic acid |  | 6 | Neg | 1 | 0 |
| 32 | Generalized skin eruption due to drugs and medicaments taken internally | Clindamycin | 6 |  | Pos | 1 | 1 |
| 33 | Rash and other nonspecific skin eruptions | Sulfamethoxazole and trimethoprim | 5 |  | Pos | 6 | 2 |
|  |  | Micafungin | 5 |  | Neg |  |  |
|  |  | Teicoplanin | 5 |  | Pos |  |  |
|  |  | Ganciclovir | 5 |  | Neg |  |  |
|  |  | Cefotaxime | 5 |  | ND |  |  |
|  |  | Meropenem | 5 |  | Neg |  |  |
| 33 | Tubulo-interstitial nephritis | Colchicina | 6 |  | Neg | 1 | 0 |
| 34 | Rash and other nonspecific skin eruptions | Sulfamethoxazole | 4 |  | Neg | 2 | 1 |
|  |  | Trimethoprim | 4 |  | Pos |  |  |
| 34 | Rash and other nonspecific skin eruptions | Amoxicillin | 6 |  | ND | 1 | ND |
| 34 | Rash and other nonspecific skin eruptions | Clindamycin | 6 |  | Pos | 1 | 1 |
| 35 | Agranulocytosis | Amoxicillin | 6 |  | Pos | 1 | 1 |
| 36 | Drug-induced liver injury | Comirnaty vaccine |  | 7 | Pos | 3 | 3 |
|  |  | PEG 2000 |  | 7 | Pos |  |  |
|  |  | PS 80 |  | 7 | Pos |  |  |
| 37 | Urticaria | Amoxicillin/Clavulanic acid | 9 |  | Pos | 1 | 1 |
| 38 | Tubulo-interstitial nephritis | Ibuprofen | 4 |  | Neg | 4 | 2 |
|  |  | Paracetamol | 4 |  | Neg |  |  |
|  |  | Cefuroxime | 5 |  | Pos |  |  |
|  |  | Ciprofloxacin | 5 |  | Pos |  |  |
| 39 | Generalized skin eruption due to drugs and medicaments taken internally | Amoxicillin | 10 |  | Pos | 1 | 1 |
| 40 | Rash and other nonspecific skin eruptions | Paracetamol | 7 |  | Pos | 1 | 1 |
| 41 | Drug-induced acute pancreatitis | Valproic acid | 7 |  | Pos | 1 | 1 |
| 42 | Tubulo-interstitial nephritis | Metamizole | 6 |  | Pos | 1 | 1 |
| 43 | Generalized skin eruption due to drugs and medicaments taken internally | Ibuprofen | 8 |  | Pos | 2 | 2 |
|  |  | Paracetamol | 8 |  | Pos |  |  |
| 44 | Localized skin eruption due to drugs and medicaments taken internally | Paracetamol | 6 |  | Neg | 1 | 0 |
| 45 | Eosinophilia | Meropenem | 4 |  | Neg | 2 | 0 |
|  |  | Fluconazole | 4 |  | Neg |  |  |
| 46 | Localized skin eruption due to drugs and medicaments taken internally | Amoxicillin/Clavulanic acid | 7 |  | Pos | 4 | 1 |
|  |  | Metamizole | 2 |  | Neg |  |  |
|  |  | Ibuprofen | 2 |  | Neg |  |  |
|  |  | Paracetamol | 0 |  | Neg |  |  |
| 47 | Agranulocytosis | Paracetamol | 3 |  | Neg | 1 | 0 |
| 48 | Localized skin eruption due to drugs and medicaments taken internally | Sulfamethoxazole and Trimethoprim | 6 |  | Pos | 1 | 1 |
| 49 | Generalized skin eruption due to drugs and medicaments taken internally | Ibuprofen | 6 |  | Neg | 2 | 0 |
|  |  | Paracetamol | 6 |  | Neg |  |  |
| 50 | Drug-induced liver injury | Amoxicillin/Clavulanic acid |  | 8 | Pos | 5 | 3 |
|  |  | Levetiracetam |  | 8 | Pos |  |  |
|  |  | Propofol |  | 8 | Pos |  |  |
|  |  | Erythromycin |  | 8 | Neg |  |  |
|  |  | Piperacillin + tazobactam |  | 8 | Neg |  |  |
| 51 | Drug rash with eosinophilia and systemic symptoms syndrome | Amoxicillin/Clavulanic acid | 6 |  | Neg | 1 | 0 |
| 52 | Urticaria | Amoxicillin | 7 |  | Neg | 1 | 0 |
| 53 | Localized skin eruption due to drugs and medicaments taken internally | Amoxicillin/Clavulanic acid | 6 |  | Neg | 1 | 0 |
| 54 | Localized skin eruption due to drugs and medicaments taken internally | Levetiracetam | 5 |  | Pos | 2 | 2 |
|  |  | Topiramate | 5 |  | Pos |  |  |
| 54 | Localized skin eruption due to drugs and medicaments taken internally | Levetiracetam | 6 |  | Pos | 2 | 1 |
|  |  | Topiramate | 6 |  | Neg |  |  |
| 55 | Druginduced liver injury | Paracetamol |  | 3 | Neg | 2 | 0 |
|  |  | Ibuprofen |  | 5 | Neg |  |  |
| 56 | Drug-induced liver injury | Ceftriaxone |  | 8 | Neg | 2 | 0 |
|  |  | Ranitidine |  | 5 | Neg |  |  |
| 57 | Agranulocytosis | Vancomycin | 6 |  | Neg | 2 | 0 |
|  |  | Teicoplanin | 6 |  | Neg |  |  |
| 58 | Acute myocarditis | PEG 2000 | 6 |  | Neg | 2 | 0 |
|  |  | PS 80 | 6 |  | Neg |  |  |
| 59 | Acute myocarditis | PEG 2000 | 6 |  | Neg | 2 | 0 |
|  |  | PS 80 | 6 |  | Neg |  |  |
| 60 | Encephalitis, myelitis and encephalomyelitis | PEG 2000 | 4 |  | Neg | 2 | 0 |
|  |  | PS 80 | 4 |  | Neg |  |  |
| 61 | Aplastic anaemia | PEG 2000 | 3 |  | Neg | 2 | 0 |
|  |  | PS 80 | 3 |  | Neg |  |  |
| 62 | Thrombocytopenia | Azithromycin | 6 |  | Pos | 2 | 2 |
|  |  | Fluconazole | 6 |  | Pos |  |  |
| 62 | Drug-induced liver injury | Azithromycin |  | 5 | Pos | 2 | 2 |
|  |  | Fluconazole |  | 5 | Pos |  |  |
| 63 | Rash and other nonspecific skin eruptions | Oxcarbazepine | 6 |  | Pos | 1 | 1 |
| 64 | Generalized skin eruption due to drugs and medicaments taken internally | Amoxicillin | 10 |  | Pos | 1 | 1 |
| 65 | Urticaria | Other antiviral vaccines | 5 |  | Pos | 1 | 1 |
| 66 | Rash and other nonspecific skin eruptions | Amoxicillin | 5 |  | Pos | 1 | 1 |
| 67 | Rash and other nonspecific skin eruptions | Cefotaxime | 4 |  | Pos | 2 | 1 |
|  |  | Clindamycin | 4 |  | Neg |  |  |
| 68 | Encephalitis, myelitis and encephalomyelitis | Naproxen | 7 |  | Neg | 3 | 0 |
|  |  | Diazepam | 5 |  | Neg |  |  |
|  |  | Tramadol | 5 |  | ND |  |  |
| 69 | Agranulocytosis | Metamizole | 6 |  | Pos | 1 | 1 |
| 70 | Angioneurotic oedema | Naproxen | 6 |  | Pos | 1 | 1 |
| 71 | Agranulocytosis | Omeprazole | 5 |  | Neg | 2 | 0 |
|  |  | Ibuprofen | 5 |  | Neg |  |  |
| 72 | Generalized skin eruption due to drugs and medicaments taken internally | Amoxicillin | 3 |  | ND | 4 | 1 |
|  |  | Paracetamol | 5 |  | Neg |  |  |
|  |  | Dexchlorpheniramine | 4 |  | Pos |  |  |
|  |  | Ibuprofen | 4 |  | Neg |  |  |
| 73 | Generalized skin eruption due to drugs and medicaments taken internally | Amoxicillin | 6 |  | Pos | 1 | 1 |
| 74 | Drug-induced acute pancreatitis | Naproxen | 7 |  | Neg | 2 | 1 |
|  |  | Ibuprofen | 7 |  | Pos |  |  |
| 75 | Drug-induced liver injury | Paracetamol |  | 8 | Neg | 1 | 0 |
| 76 | Agranulocytosis | Acetazolamide | 2 |  | ND | 2 | 1 |
|  |  | Cefotaxime | 7 |  | Pos |  |  |
| 77 | Druginduced acute pancreatitis | Mesalazine | 7 |  | Neg | 1 | 0 |
| 78 | Agranulocytosis | Mycophenolate mofetil | 5 |  | Neg | 3 | 0 |
|  |  | Valganciclovir | 6 |  | Neg |  |  |
|  |  | Cefixime | 6 |  | Neg |  |  |
| 79 | Generalized skin eruption due to drugs and medicaments taken internally | Amoxicillin | 4 |  | Neg | 1 | 0 |
| 80 | Urticaria | Tramadol | 5 |  | ND | 2 |  |
|  |  | Paracetamol | 5 |  | ND |  |  |
| 80 | Urticaria | Dexketoprofen | 6 |  | ND | 1 |  |
| 80 | Urticaria | Rifampicin | 6 |  | ND | 1 |  |
| 80 | Urticaria | Levofloxacin | 6 |  | Neg | 2 | 0 |
|  |  | Sulfamethoxazole and trimethoprim | 6 |  | Neg |  |  |
| 81 | Drug-induced liver injury | Guaifenesin |  | 6 | Pos | 3 | 1 |
|  |  | Paracetamol |  | 4 | Neg |  |  |
|  |  | Dextromethorphan |  | 5 | Neg |  |  |
|  |  |  |  |  |  |  |  |
| ND, Not Done | | | | | | | |
